# Treatment with Tie2-siRNA in combination with carboplatin suppresses the growth of Ishikawa human endometrial carcinoma cell xenografts *in vivo*

**DOI:** 10.3892/ol.2013.1295

**Published:** 2013-04-08

**Authors:** FEIFEI GUO, QINGYING XUN, HUAIJUN ZHOU

**Affiliations:** 1 Department of Gynecology and Obstetrics, Nanjing Drum Tower Hospital, Nanjing University Medical School, Nanjing, Jiangsu, P.R. China; 2 Department of Physiology, Medical College, Southeast University, Nanjing, Jiangsu, P.R. China

**Keywords:** tyrosine kinase receptor 2, siRNA, endometrial carcinoma, nude mice, carboplatin

## Abstract

It is well-known that tumor angiogenesis is important in cancer development, and studies on blocking angiogenesis to treat tumors have become one of the most promising and active fields in anticancer research. The present study investigated the effect of siRNA targeting the tyrosine kinase receptor 2 (Tie2) gene in combination with carboplatin in a mouse model of endometrial carcinoma in an attempt to elucidate the role of Tie2 in the carcinogenesis and progression of endometrial carcinoma via angiogenesis, in order to establish a basis for the development of complementary molecule targeting and chemotherapeutic actions. Ishikawa cells were used to establish a human endometrial carcinoma nude mouse tumor xenograft model. Tie2-siRNA (20 *μ*g/mouse) and/or carboplatin (25.0 mg·kg^−1^) were administered as the treatment strategy. Real-time PCR and western blotting were used to evaluate the expression levels of Tie2 mRNA and protein and immunohistochemistry was used to assess the vessel density of the tumor tissues. The present data demonstrated that Tie2-siRNA and/or carboplatin were able to suppress the growth of endometrial xenografts *in vivo* and attenuate the expression of Tie2 mRNA and protein, as assessed by real-time PCR and western blotting. Furthermore, immunohistochemical assessment showed that the vessel density of the tumors decreased with treatment. The present results suggest that treatment with Tie2-siRNA or carboplatin alone was able to inhibit the growth of human endometrial carcinoma nude mouse xenografts markedly and decrease the expression of Tie2. The combination of Tie2-siRNA and carboplatin increased the therapeutic effect of carboplatin which may eliminate the tumor microenvironment, increase the apoptosis of tumor cells, normalize the abnormal tumor vessels and increase the efficiency of chemotherapy for endometrial carcinoma with carboplatin. The synergy of Tie2-siRNA in combination with carboplatin may involve the regulation of other angiogenesis and metastasis pathways.

## Introduction

Endometrial carcinoma affects older females, with ∼75% of the cases occurring after the onset of the menopause, and is the third most common gynecological malignant tumor. Its incidence has increased markedly and it is estimated that ∼43,470 new cases of endometrial carcinoma were diagnosed and 7,950 mortalities occurred in the USA in 2010 (1,2). Surgery, chemotherapy and radiation approaches (3,4) have been established for the treatment of endometrial carcinoma. However, the recurrent cases that have acquired radio- or chemoresistance pose a major challenge for healthcare professionals. It is therefore necessary to identify new, effective and comprehensive treatments.

Angiogenesis is a physiological process that normally occurs in fetal development, wound healing and in the female reproductive tract. It has been reported that the growth of new blood vessels has pathological and beneficial roles in human diseases and the growth of tumors also relies on adequate blood supplies. Angiogenesis is considered to be crucial for tumor malignant biological characteristics, such as invasion, recurrence and metastasis (2,5) and its importance in solid tumor growth and metastasis has been widely recognized by multiple studies. Studies concerning blocking angiogenesis to treat tumors have drawn much attention and become one of the most promising and active fields in anticancer research (6–9). The present study investigated the effect of siRNA targeted against the tyrosine kinase receptor 2 (Tie2) gene in combination with carboplatin in a mouse model of endometrial carcinoma in an attempt to elucidate the role of Tie2 in the carcinogenesis and progression of endometrial carcinoma via angiogenesis, in order to establish a basis for the development of complementary molecule targeting and chemotherapeutic actions.

## Materials and methods

### Cell culture

Ishikawa cells, a human endometrial carcinoma cell line, were generously provided as a gift by Professor L.H. Wei (Peking University People’s Hospital, Beijing, China) and cultured in α-Modified Eagle’s medium (α-MEM; Gibco, Carlsbad, CA, USA) supplemented with 10% heat-inactived fetal bovine serum (FBS, Gibco), penicillin (100 IU/ml) and streptomycin (100 *μ*g/ml). All cultures were incubated in a 5% CO_2_ humidified incubator at 37°C and experiments were performed using subconfluent cells in the exponential growth phase.

### Nude mouse tumor xenograft model

The animal care procedures conformed with the institutional guidelines in compliance with the national and international laws and policies. Female 3–4-week-old athymic nude mice (BALB/c-nu/nu; n=25), obtained from Shanghai Animal Center (Shanghai, China), were used for all experiments. The mice were housed and maintained in laminar flow cabinets under specific pathogen-free conditions.

Following the construction of the nude mouse tumor xenograft model with Ishikawa cells, all the injected nude mice with tumors were randomly divided into five groups, with five mice in each group as follows, when the volume of the xenografts reached 50 mm^3^ (∼2 weeks): i) blank (5% glucose; G) group; ii) pRNAT-CMV3.2-Neo carrying negative siRNA (N) group; iii) carboplatin (C) group; iv) pRNAT-CMV3.2-Neo carrying Tie2-siRNA (T) group; v) Tie2-siRNA in combination with carboplatin (A) group. The five groups received intra-tumor injections of 5% glucose (0.2 ml·mouse^−1^), N-siRNA (20 *μ*g·mouse^−1^), carboplatin (25.0 mg·kg^−1^, 5% glucose dilution), Tie2-siRNA (20 *μ*g·mouse^−1^) and Tie2-siRNA (20 *μ*g·mouse^−1^) in combination with carboplatin (25.0 mg·kg^−1^, 5% glucose dilution), respectively, every three days for five cycles. The grafts were measured with a sliding caliper and the tumor sizes were caculated using the equation: tumor size =[length (mm) × width (mm)^2^] / 2. The inhibitory rates of the primary tumors were calculated as: (Tumor size_control_ − tumor size_treatment_) / tumor size_control_ ×100. All mice were sacrificed following the last administration.

### Real-time PCR assay

The total RNA was isolated from the tumor tissue samples using TRIzol reagent (Invitrogen, Carlsbad, CA, USA) and the amount and purity of the extracted RNA was quantitated by absorbance analysis at 260 nm using an Eppendorf biophotometer (Hamburg, Germany). The total RNA was reverse transcribed using murine leukemia virus reverse transcriptase (Takara, Ōtsu, Japan). GAPDH primer was used as an internal standard. PCR reactions were performed using the SYBR PrimeScript RT-PCR kit (Takara) and the following primers: for Tie2: forward, 5′-GTTCTGTCTCCCTGACCCCTAT-3′ and reverse, 5′-TAAGCTTACAATCTGGCCCGTA-3′; and for GAPDH, forward, 5′-ATTCCATGGCACCGTCAAGGCTG-3′ and reverse, 5′-GTGGTGAAGACGCCAGTGGACT-3′. To ensure the specificity of the Tie2 primer set, the amplicons generated from the PCR reactions were evaluated in terms of specific melting point temperatures using the first derivative primer melting curve software (Applied Biosystems, Foster City, CA, USA). The expression level of Tie2 mRNA was normalized to the expression of the control gene GAPDH and the relative quantification of Tie2 mRNA was performed using the comparative cycle threshold method (2^−ΔΔCT^) (10). All PCR experiments were repeated three times.

### Western blot analysis

The tumor tissue samples were homogenized in RIPA buffer with a protease and phosphatase cocktail (Roche, Mannheim, Germany). The proteins underwent 10% SDS-PAGE and were then transferred onto nitrocellulose membranes (Millipore, Billerica, MA, USA). After incubation with the primary antibody (1:1,000; CST) at 4°C overnight, the membranes were washed with Tris-buffered saline Tween-20 (TBST), then incubated with a goat anti-mouse-HRP secondary antibody (1:5,000 dilution, Abcam, Cambridge, MA, USA) and visualized using ECL chemoluminescence (Millipore). GAPDH (Abcam) was used as an internal standard. The relative intensity of the target blots was analyzed using Quantity One software (Bio-Rad, Hercules, CA, USA).

### Immunohistochemical assessment of vessel density

The tumor tissues were paraffin-embedded and sectioned (4-*μ*m thickness) after being fixed in formalin for 24 h. The slides were deparaffined and microwaved at 98°C for 10 min in citrate buffer, then rehydrated through descending grades of ethanol. Endogenous peroxidase activity was quenched by immersion in 3% H2O_2_ for 10 min. The sections were then blocked with 10% FBS (Gibco) and incubated with appropriately diluted (1:150) rabbit CD34 monoclonal antibody (one type of blood vessel marker; Abcam) at 4°C overnight. The primary antibody was removed and washed with TBS and FITC-conjugated goat-anti-rabbit IgG antibody (Zhongshan Bio-tech Co., Ltd., Zhongshan, China) was then added, incubated at 37°C for 30 min. The sections were then visualized using DAB coloration fluid (Zhongshan Bio-tech Co., Ltd.). Finally, the sections were stained with hematoxylin and washed with distilled water. Quantification of blood vessels was performed as described previously (11). A negative control was included for each tissue section by substituting the primary antibody for a matching concentration of goat or rabbit IgG.

The brown-stained single endothelial cell or cell clusters distinct from adjacent microvessels, tumor cells or other stromal cells were considered as single countable microvessels. The areas with the highest microvessel density (MVD) were identified in a low-power field (magnification, ×100) and vessels were counted in five high-power fields (magnification, ×200). The average numbers of microvessels were presented as the mean ± SEM. To minimize subjectivity, the process was performed by blinded pathologists.

### Statistical analysis

All values were presented as the mean ± SEM and analyzed for significance by one-way analysis of variance (ANOVA). The SPSS 16 statistical software (SPSS, Chicago, IL, USA) was used for the analyses. P<0.05 was considered to indicate statistically significant differences.

## Results

### Difference in tumor growth following treatment with Tie2-siRNA and/or carboplatin

**Figure 1:**
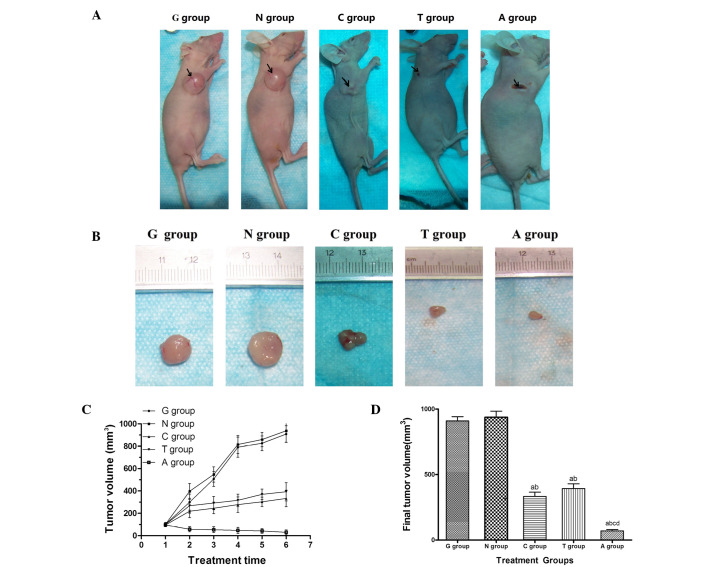
Tie2-siRNA and/or carboplatin affects tumor growth *in vivo*. (A) Macroscopic appearance of tumors in nude mice 30 days after the transplantation of human endometrial carcinoma Ishikawa cells. Arrows outline the representative tumors in the right scapular region of the mice. (B) Images of representative excised tumors from each group. (C) Tumor volumes were averaged for each treatment group and time point over the course of the study. (D) Final tumor volumes of the isolated tumors were averaged for each group (mean ± SEM). The tumor volumes of mice treated with of carboplatin, Tie2-siRNA and the combined administration were significantly different, compared with the G or N group, beginning at the third treatment and continuing to be significant throughout the study (P<0.05 respectively). ^a^Compared with G group, there was a statistically significant difference; ^b^Compared with N group, there was a statistically significant difference; ^c^Compared with C group, there was a statistically significant difference; ^d^Compared with T group, there was a statistically significant difference. Tie2, tyrosine kinase receptor 2; G, glucose treatment; N, pRNAT-CMV3.2-Neo carrying negative siRNA treatment; C, carboplatin treatment; T, pRNAT-CMV3.2-Neo carrying Tie2-siRNA treatment; A, Tie2-siRNA in combination with carboplatin treatment.

The effect of Tie2 gene silencing with or without carboplatin was investigated in the Ishikawa cell tumor xenograft model *in vivo*. The control and experimental mice developed tumors at the site of injection. Typically, 10–14 days were required after cell transplantation for the volume of the xenograft to reach 100 mm^3^. The representative images of mice (outline with arrow, Fig. 1A) and excised tumors (Fig. 1B) from each group are shown in Fig. 1A and B. The mice which received intratumor treatment with carboplatin, Tie2-siRNA and combined therapy exhibited reductions in tumor size in comparison with the G or N group mice. The treatment had significant effects on tumor growth beginning on the second treatment and continued to be significant throughout the study (Fig. 1C). During the treatment period, the tumor growth curves were essentially the same in the G and N group mice (Fig. 1C). However, while carboplatin and Tie2-siRNA significantly slowed tumor growth (Fig. 1C), the combination of carboplatin and Tie2-siRNA resulted in a greater retardation of tumor growth than single administrations (Fig. 1C). The final average tumor volumes were smaller in the carboplatin (332.99±73.91 mm^3^), Tie2-siRNA (392.78±81.74 mm^3^) and combined administration (70.11±22.09 mm^3^) groups than those in the G (909.05±73.42 mm^3^) or N (937.65±103.09 mm^3^) groups, and the differences were statistically significant (Fig. 1D). The combined administration was significantly different compared with the carboplatin and Tie2-siRNA groups (Fig. 1D). The mean tumor inhibition rates in the carboplatin, Tie2-siRNA and combined administration groups were 62.91±4.50, 75.18±8.39 and 85.93±5.35%, respectively compared with those of the G and N (0.8±5.86%) groups, and the differences were statistically significant.

### Difference in Tie2 mRNA expression following treatment with Tie2-siRNA and/or carboplatin

**Figure 2:**
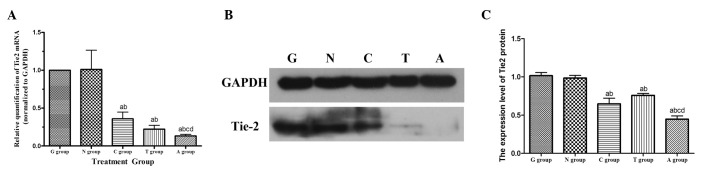
Tie2-siRNA and/or carboplatin decreases Tie2 expression in the tumor tissues of the groups. (A) Total RNA was isolated from tumor tissues and relative Tie2 mRNA expression was analyzed with real-time PCR. (B) Western blot analysis of Tie2 protein expression in five groups and (C) quantification of relative Tie2 protein expression tumor tissues of every group (P<0.05). ^a^Compared with G group, there was a statistically significant difference; ^b^Compared with N group, there was a statistically significant difference; ^c^Compared with C group, there was a statistically significant difference; ^d^Compared with T group, there was a statistically significant difference. Tie2, tyrosine kinase receptor 2; G, glucose treatment; N, pRNAT-CMV3.2-Neo carrying negative siRNA treatment; C, carboplatin treatment; T, pRNAT-CMV3.2-Neo carrying Tie2-siRNA treatment; A, Tie2-siRNA in combination with carboplatin treatment.

The antitumor treatment had significant effects on tumor growth in the present study and subsequently Tie2 gene expression was investigated by real-time PCR. The amount of Tie2 mRNA was normalized to the expression of the control gene GAPDH and relative quantification was performed using the 2^−ΔΔCT^ method. The expression of Tie2 mRNA was significantly reduced following treatment with carboplatin (0.36±0.23), Tie2-siRNA (0.22±0.14) and the combined administration of the two (0.13±0.05) compared with the G (1.00±0.00) or N (1.01±0.67) groups. Compared with the administration of carboplatin or Tie2-siRNA alone, the reduction of Tie2 mRNA expression level in the the combined administration group was statistically significant (P<0.05; Fig. 2A).

### Difference in expression level of Tie2 protein following treatment with Tie2-siRNA and/or carboplatin

Since the expression of Tie2 mRNA was significantly reduced following treatment with Tie2-siRNA and/or carboplatin, the effects of this treatment on Tie2 protein expression levels in the present treatment groups was investigated next. Xenograft homogenates were analyzed by western blotting and probed with anti-Tie2 antibody and GAPDH was used as a loading control. The results of the blotting revealed that the Tie2 protein levels were significantly reduced in the C (0.805±0.085), T (0.670±0.109) and A (0.590±0.135) groups compared with the G (1.059±0.085) or N (1.018±0.069) groups (Fig. 2B and C). The protein level in the A group exhibited the most marked reduction and was significantly different compared with treatment using carboplatin or Tie2-siRNA alone (P<0.05). Taken together with the real-time PCR data, it may be suggested that silencing the Tie2 gene enhances the the antitumor activity of carboplatin. This may be through the normalization of tumor vessels during the chemotherapy period.

### Differences in tumor vascularity following treatment with Tie2-siRNA and/or carboplatin

**Figure 3:**
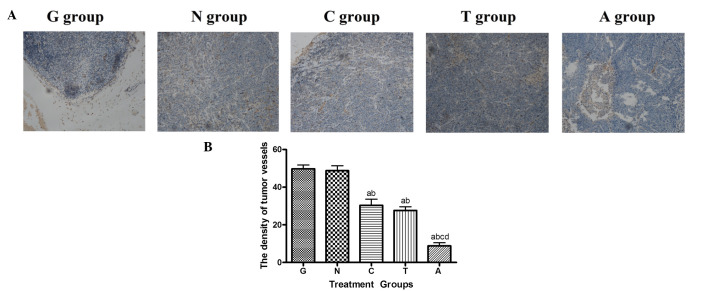
Inhibition of tumor angiogenesis following treatment. (A) Immunohistochemical staining of tumor xenografts probed with anti-CD34 antibody after adminastration to visualize endothelial cells and tumor vascular formation. The images of the microvessel density and vessel characteristics of the treatment groups are shown (magnification, ×200). (B) Results of immunohistochemical staining. The densitiy of tumor vessels were lower in the carboplatin, Tie2-siRNA and the combined administration groups than those in the G and N groups (P<0.05). The combined administration group showed the greatest reduction in the number of tumor vessels and the reduction was significantly different compared with the other groups (P<0.05). ^a^Compared with G group, there was a statistically significant difference; ^b^Compared with N group, there was a statistically significant difference; ^c^Compared with C group, there was a statistically significant difference; ^d^Compared with T group, there was a statistically significant difference. Tie2, tyrosine kinase receptor 2; G, glucose treatment; N, pRNAT-CMV3.2-Neo carrying negative siRNA treatment; C, carboplatin treatment; T, pRNAT-CMV3.2-Neo carrying Tie2-siRNA treatment; A, Tie2-siRNA in combination with carboplatin treatment.

The growth of new blood vessels has an important role in tumor progression. To determine whether Tie2 siRNA-mediated downregulation of Tie2 was involved in the inhibition of angiogenesis, the density of tumor vessels was investigated by immunohistochemical assessment and probed with anti-CD34 antibody. The expression of CD34 in the tumor tissues of T (27.60±4.56) group, C (35.80±2.17) and A (15.40±2.07) group was poor, but was high in the G (49.80±4.44) and N groups (48.80±5.81). Microvessel counting showed that the MVD was higher in the G and N groups compared with the T group (Fig. 3A).

## Discussion

Angiogenesis is a key element for the development of tumors as it provides new blood supplies and subsequently allows malignant progression. This suggests a new strategy for the treatment of carcinoma. The critical role of angiogenesis in ovarian and endometrial function has been demonstrated previously (12). During tumor angiogenesis, vascular quiescence and stabilization are regulated by different signal molecules. It is generally accepted that tumor angiogenesis is the result of unbalanced expression of angiogenic factors.

Angiogenesis also involves an extremely complicated network and is modulated by numerous growth factors. Vascular endothelial growth factor (VEGF), expressed predominantly on vascular endothelial cells, is one of the most important regulators of vascularization and an attractive target for anti-angiogenic therapy. VEGF is the most importaint angiogenic regulator that increases vascular permeability and promotes endothelial proliferation (12). Agents such as endostatin and anti-VEGF antibodies that inhibit the VEGF receptor have been developed and may result in effective inhibition of solid tumor growth *in vivo*(13–16). However, VEGF-targeted therapy is restricted by its transient responses in the clinic due to drug resistance and it has been reported that the agents were not effective for all tumor types, indicating that blocking VEGF activation alone may not be sufficient to completely halt tumor angiogenesis, since angiopoietin and other factors are also involved.

Tie and angiopoietin (Ang) are involved in another signaling system which is not only crucial for angiogenesis and vascular homeostasis, but is also vital in the progression of numerous types of cancer (17–19). Ang1 is produced by pericytes and other cells, whereas Ang2 and Tie2 are expressed mainly on endothelial cells. Tie2 was originally identified as the second member of an orphan RTK subfamily and is an Ang-specific receptor with a critical role in the modulation of vascular generation and remodeling (20–22). It has been reported that Tie2 is critical in tumor-induced angiogenesis (23). The suppression of Tie2 signaling caused by the use of specific blocking agents may be able to suppress the growth of tumors and several studies have shown that interfering with the Tie2 receptor pathway resulted in the inhibition of tumor angiogenesis and growth (24–27). The expression of Tie2 in tumor vessels may also indicate a role for Tie2 in tumor angiogenesis and the pathological angiogenesis contributing to the progression of diseases. Thus this strategy may be used for anti-angiogenesis treatment in anticancer therapy. The Tie/Ang system is important for endometrial vessel development during the postovulatory phase and has a critical association with the initiation of endometrial diseases. Thus the potential for inhibiting angiogenesis is likely to have implications for the treatment of endometrial cancer.

Chemotherapy is an important and complementary treatment for advanced and recurrent patients with endometrial cancer and platinum-based chemotherapy has been an important treatment for these patients. Carboplatin is cis-diammine (1,1-cyclobutanedicarboxylate) platinum which has an effective activity against human tumors. It has little renal toxicity, ototoxicity or neurotoxicity compared with cisplatin in clinical studies. Early studies have shown that carboplatin acts through the inhibition of DNA replication and transcription and by shielding the repair of damaged DNA. However, the mechanisms of carboplatin action may be more complicated, and research concerning the anticancer mechanism of carboplatin is scarce.

The present study aimed to elucidate the role of Tie2 in the carcinogenesis and progression of endometrial carcinoma via angiogenesis, with a focus on establishing a basis for the development of complementary molecule targeting action. The experiments showed that the treatment of endometrial cacinoma in nude mice with carboplatin and Tie2-siRNA, alone or in combination, resulted in delays in tumor growth and reductions in tumor size and vascularity (Figs. 1 and 3). Tie2 expression was significantly silenced in the tumor tissues of the combined therapy, Tie2-siRNA and carboplatin treatment groups, which was consistent with previous studies (28). This may indicate that Tie2 is important in the carcinogenesis and progression of cancer via angiogenesis.

It is well known that tumor vessels are structurally and functionally abnormal. Tumor vessels may be tortuous and leaky, lacking the hierarchical arrangement of arterioles, capillaries and venules and existing in a constantly dynamic state, which becomes increasingly resistent to conventional chemotherapy due to the slow proliferation of cells (29). The vessel number and normalized tumor vessels are enough to maintain tumor growth (20). Current anticancer therapies use normalized tumor vessels to deliver chemotherapeutics or other cancer cell-targeting drugs more efficiently (30). In the present study, during the combined administration, the expression of Tie2 was silenced first in order to normalize the tumor vascularity, followed by the administration of the chemotherapeutic drug carboplatin, so carboplatin was able to affect all areas of the tumors. The combined administration exhibited more noticeable effects in delaying tumor growth and reducing tumor size and vascularity compared with carboplatin or Tie2-siRNA administration alone, and the difference was significant. This was in accordance with previous studies (31) and also showed a role for Tie2 in tumor angiogenesis.

The growth and maintenance of tumor blood vessels depend on multiple growth factors. Making use of the effect of the interplay among the growth factors is required in order to advance growth factor-targeted cancer therapies. Thus, agents that block multiple angiogenesis targets are being developed for the treatment of tumors. In the present study, a nude mouse endometrial carcinoma model was successfully constructed and the critical role of the Tie2 gene in tumor angiogenesis and endometrial carcinoma was demonstrated. It is the first time that the inhibition of the Tie2 gene has been used in combination with carboplatin to suppress tumor growth in endometrial carcinoma by interrupting tumor angiogenesis in xenograft models. The present findings revealed that local administration of Tie2-siRNA is able to mediate effective knockdown of the target protein and suppress tumor growth *in vivo*. Together with the other results of the present study, this may suggest that intratumor injections of pRNAT-CMV3.2-Tie2 may silence the expression of Tie2, subsequently eliminate the tumor angiogenesis and metastasis pathway regulated by Tie2 and increase the efficiency of chemotherapy for endometrial carcinoma using carboplatin. However, siRNAs are not an optimal treatment due to their short half lives and transient effects (32,33). Other anti-Tie2 treatment modalities more suitable for therapy, such as modified RNAi or an antibody, should be developed in the future. At present, little is known with regard to the abnormal stucture and function and the hierarchical arrangement of arterioles, capillaries and venules of tumor vessels. Microangiography may be useful for structural and functional studies of tumor vessels in future. In summary, the findings of the present study reveal a new direction for the rational design and scheduling of siRNA-based molecular targeting therapeutic strategies in combination with anti-angiogenic drugs and/or other therapies.

## References

[1] National Cancer Institute at the National Institutes of Health Endometrial Cancer Home Page. http://seer.cancer.gov/statfacts/html/corp.html.

[2] Jemal A, Bray F, Center MM, Ferlay J, Ward E, Forman D (2011). Global cancer statistics. CA Cancer J Clin.

[3] Myatt SS, Wang J, Monteiro LJ, Christian M, Ho KK, Fusi L, Dina RE, Brosens JJ, Ghaem-Maghami S, Lam EW (2010). Definition of microRNAs that repress expression of the tumor suppressor gene FOXO1 in endometrial cancer. Cancer Res.

[4] Ray M, Fleming G (2009). Management of advanced-stage and recurrent endometrial cancer. Semin Oncol.

[5] Poon RT, Lau CP, Ho JW, Yu WC, Fan ST, Wong J (2003). Tissue factor expression correlates with tumor angiogenesis and invasiveness in human hepatocellular carcinoma. Clin Cancer Res.

[6] Carmeliet P (2005). Angiogenesis in life, disease and medicine. Nature.

[7] Sistla A, Kertelj A, Shenoy N (2008). Development of an intravenous formulation of SU010382 (prodrug of SU5416, an anti-angiogenesis agent). PDA J Pharm Sci Technol.

[8] Griffioen AW (2008). Anti-angiogenesis: making the tumor vulnerable to the immune system. Cancer Immunol Immunother.

[9] Kimura Y, Sumiyoshi M, Baba K (2008). Anti-tumor actions of major component 3′-O-acetylhamaudol of *Angelica japonica* roots through dual actions, anti-angiogenesis and intestinal intraepithelial lymphocyte activation. Cancer Lett.

[10] Schmittgen TD, Livak KJ (2008). Analyzing real-time PCR data by the comparative C(T) method. Nat Protoc.

[11] Weidner N (1995). Current pathologic methods for measuring intratumoral microvessel density within breast carcinoma and other solid tumors. Breast Cancer Res Treat.

[12] Klauber N, Rohan RM, Flynn E, D’Amato RJ (1997). Critical components of the female reproductive pathway are suppressed by the angiogenesis inhibitor AGM-1470. Nat Med.

[13] Yamaguchi R, Yano H, Nakashima Y, Ogasawara S, Higaki K, Akiba J, Hicklin DJ, Kojiro M (2000). Expression and localization of vascular endothelial growth factor receptors in human hepatocellular carcinoma and non-HCC tissues. Oncol Rep.

[14] Miller K, Wang M, Gralow J, Dickler M, Cobleigh M, Perez EA, Shenkier T, Cella D, Davidson NE (2007). Paclitaxel plus bevacizumab versus paclitaxel alone for metastatic breast cancer. N Engl J Med.

[15] Gerber HP, Ferrara N (2005). Pharmacology and pharmacodynamics of bevacizumab as monotherapy or in combination with cytotoxic therapy in preclinicals studies. Cancer Res.

[16] Ferrara N, Kerbel RS (2005). Angiogenesis as a therapeutic target. Nature.

[17] Sandler A, Gray R, Perry MC, Brahmer J, Schiller JH, Dowlati A, Lilenbaum R, Johnson DH (2006). Paclitaxel-carboplatin alone or with bevacizumab for non-small-cell lung cancer. N Engl J Med.

[18] Sugimachi K, Tanaka S, Taguchi K, Aishima S, Shimada M, Tsuneyoshi M (2003). Angiopoietin switching regulates angiogenesis and progression of human hepatocellular carcinoma. J Clin Pathol.

[19] Hu B, Guo P, Fang Q, Tao HQ, Wang D, Nagane M, Huang HJ, Gunji Y, Nishikawa R, Alitalo K, Cavenee WK, Cheng SY (2003). Angiopoietin-2 induces human glioma invasion through the activation of matrix metalloprotease-2. Proc Natl Acad Sci USA.

[20] Yang H, Yang K, Hu JK, Tang H, Zhang B, Chen ZX, Wang YJ, Chen JP (2010). Eukaryotic expression of extracellular ligand binding domains of murine Tie-2 and its anti-angiogenesis effect in SGC-7901 cell lines. J Gastroenterol Hepatol.

[21] Dumont DJ, Yamaguchi TP, Conlon RA, Rossant J, Breitman ML (1992). tek, a novel tyrosine kinase gene located on mouse chromosome 4, is expressed in endothelial cells and their presumptive precursors. Oncogene.

[22] Sato TN, Tozawa Y, Deutsch U, Wolburg-Buchholz K, Fujiwara Y, Gendron-Maguire M, Gridley T, Wolburg H, Risau W, Qin Y (1995). Distinct roles of the receptor tyrosine kinases Tie-1 and Tie-2 in blood vessel formation. Nature.

[23] Suri C, Jones PF, Patan S, Bartunkova S, Maisonpierre PC, Davis S, Sato TN, Yancopoulos GD (1996). Requisite role of angiopoietin-1, a ligand for the TIE2 receptor, during embryonic angiogenesis. Cell.

[24] Eklund L, Olsen BR (2006). Tie receptors and their angiopoietin ligands are context-depedent regulators of vascular remodeling. Exp Cell Res.

[25] Mori Y, Sahara H, Matsumoto K, Takahashi N, Yamazaki T, Ohta K, Aoki S, Miura M, Sugawara F, Sakaguchi K, Sato N (2008). Downregulation of Tie2 gene by a novel antitumor sulfolipid, 3′-sulfoquinovosyl-1′-monoacylglycerol, targeting angiogenesis. Cancer Sci.

[26] Mai J, Song S, Rui M, Liu D, Ding Q, Peng J, Xu Y (2009). A synthetic peptide mediated active targeting of cisplatin liposomes to Tie2 expressing cells. J Control Release.

[27] Yamakawa D, Kidoya H, Sakimoto S, Jia W, Takakura N (2011). 2-Methoxycinnamaldehyde inhibits tumor angiogenesis by suppressing Tie2 activation. Biochem Biophys Res Commun.

[28] Tournaire R, Simon MP, le Noble F, Eichmann A, England P, Pouysségur J (2004). A short synthetic peptide inhibits signal transduction, migration and angiogenesis mediated by Tie2 receptor. EMBO Rep.

[29] Maschek G, Savaraj N, Priebe W, Braunschweiger P, Hamilton K, Tidmarsh GF, De Young LR, Lampidis TJ (2004). 2-deoxy-D-glucose increases the efficacy of adriamycin and paclitaxel in human osteosarcoma and non-small cell lung cancers *in vivo*. Cancer Res.

[30] Sennino B, Falcón BL, McCauley D, Le T, McCauley T, Kurz JC, Haskell A, Epstein DM, McDonald DM (2007). Sequential loss of tumor vessel pericytes and endothelial cells after inhibition of platelet-derived growth factor B by selective aptamer AX102. Cancer Res.

[31] Jain RK (2005). Normalization of tumor vasculature: an emerging concept in antiangiogenic therapy. Science.

[32] Devroe E, Silver PA (2004). Therapeutic potential of retroviral RNAi vectors. Expert Opin Biol Ther.

[33] Behlke MA (2008). Chemical modification of siRNAs for *in vivo* use. Oligonucleotides.

